# BMI1 Polycomb Group Protein Acts as a Master Switch for Growth and Death of Tumor Cells: Regulates TCF4-Transcriptional Factor-Induced BCL2 Signaling

**DOI:** 10.1371/journal.pone.0060664

**Published:** 2013-05-06

**Authors:** Hifzur Rahman Siddique, Aijaz Parray, Rohinton S. Tarapore, Lei Wang, Hasan Mukhtar, R. Jeffery Karnes, Yibin Deng, Badrinath R. Konety, Mohammad Saleem

**Affiliations:** 1 Department of Molecular Chemoprevention and Therapeutics, The Hormel Institute, University of Minnesota, Austin, Minnesota, United States of America; 2 Department of Dermatology, University of Wisconsin, Madison, Wisconsin, United States of America; 3 Department of Cell Death and Cancer Genetics, The Hormel Institute, University of Minnesota, Austin, Minnesota, United States of America; 4 Department of Urology, Mayo Medical School and Mayo Clinic, Rochester, Minnesota, United States of America; 5 Department of Urology, University of Minnesota, Minneapolis, Minnesota, United States of America; 6 Department of Laboratory Medicine Pathology, University of Minnesota, Minneapolis, Minnesota, United States of America; University of Kentucky College of Medicine, United States of America

## Abstract

For advanced prostate cancer (CaP), the progression of tumors to the state of chemoresistance and paucity of knowledge about the mechanism of chemoresistance are major stumbling blocks in the management of this disease. Here, we provide compelling evidence that BMI1 polycomb group protein and a stem cell factor plays a crucial role in determining the fate of tumors vis-à-vis chemotherapy. We show that progressive increase in the levels of BMI1 occurs during the progression of CaP disease in humans. We show that BMI1-rich tumor cells are non-responsive to chemotherapy whereas BMI1-silenced tumor cells are responsive to therapy. By employing microarray, ChIP, immunoblot and Luciferase reporter assays, we identified a unique mechanism through which BMI1 rescues tumor cells from chemotherapy. We found that BMI1 regulates (i) activity of TCF4 transcriptional factor and (ii) binding of TCF4 to the promoter region of anti-apoptotic *BCL2* gene. Notably, an increased TCF4 occupancy on *BCL2* gene was observed in prostatic tissues exhibiting high BMI1 levels. Using tumor cells other than CaP, we also showed that regulation of TCF4-mediated BCL2 by BMI1 is universal. It is noteworthy that forced expression of BMI1 was observed to drive normal cells to hyperproliferative mode. We show that targeting BMI1 improves the outcome of docetaxel therapy in animal models bearing chemoresistant prostatic tumors. We suggest that BMI1 could be exploited as a potential molecular target for therapeutics to treat chemoresistant tumors.

## Introduction

According to American Cancer Society, an estimated 241,740 new cases of prostate cancer (CaP) were diagnosed and 28,170 CaP patients were projected to die in the year 2012 in USA alone [1]. CaP is the second most frequently diagnosed cancer in men in the western world [2]–[3]. CaP patients (30–50%) exhibit a local or distant recurrence of disease after surgery or therapy [4]–[6]. Although castration is a common treatment option for metastatic CaP, it does not significantly prolong the survival of patients and majority of these patients progress to castration-resistant prostate cancer (CRPC). A treatment option for CRPC is cytotoxic chemotherapy; however, chemotherapy improves overall survival in such patients by only a median of 2.9 months [6]–[7]. Despite chemotherapy, CRPC patients typically show rapid progression and develop chemoresistant disease [8]–[10]. Therefore emergence of chemoresistance is considered a major hurdle in the management of CaP. The dismal outcome of the management of chemoresistant CRPC disease could also be associated to the lack of knowledge about the molecular mechanism involved in the development of chemoresistant disease.

There is increasing evidence that polycomb group (PcG) proteins, first discovered in *Drosophila* as epigenetic gene silencers of homoeotic genes, play a crucial role in cancer development and recurrence [11]. BMI1, a member of PcG family of proteins, is a marker used in stem cell biology [11]–[12]. There is an enormous body of evidence suggesting that increased expression of BMI1 could facilitate chemoresistance [11]–[12]. Recent studies show that BMI1 is positively correlated with poor prognosis in cancer patients [13]–[16]. We recently reviewed the significance of BMI1 in the emergence of chemoresistance in various types of cancers [11]. Glinsky et al. identified BMI1 as one the signature molecules in a broad spectrum of therapy-resistant cancers including CaP [17]. Except a few regulatory functions of BMI1 in cell cycle (suppressing p16INK4a and p14ARF), not much is known about it mechanism of action. In this study, we determined the relevance of BMI1 in chemoresistance of CaP and delineate its mechanism of action both *in vitro* and *in vivo*. In addition, we establish the utility of BMI1 as a molecular target for therapeutic agents to overcome chemoresistance.

## Materials and Methods

### Cell Lines and plasmids

Normal primary prostate epithelial cell (PrEC) was procured from Cambrex BioScience (Walkersville, MD, USA). Normal and transformed prostate epithelial cell line **(**RWPE1), CaP cell lines (LNCaP, 22Rν1, C42b, PC3 and Du145), prostatic stromal myofibroblasts (WPMY1), and colon cancer cell lines HT29 were obtained from ATCC (Manassas, VA, USA). LAPC4 cells were gifted by Dr. Robert Reiter (UCLA, Los Angeles, CA, USA) who generated these cells [18]. The pbabe-BMI1 plasmid was a kind gift from Dr. Chi V. Dang (The John Hopkins University, Baltimore, MD, USA). pGeneClip, pbabe plasmids and pGeneClip-BMI1-shRNA were procured from SA-Biosciences Corporation (Fredrick, MD, USA). pTK-TCF-Luc (TopFlash and FopFlash) was procured from Upstate Laboratories (Lake Placid, NY, USA). Cells were cultured in appropriate media and were kept in 5% CO_2_ in an incubator at 37°C.

### Tumor tissues

Frozen surgical prostatic tissues and tissues in paraffin blocks were procured from NCI-sponsored Cooperative Human Tissue Network (CHTN, Mid-West Division, University of Ohio, Columbus, USA). The quality of the frozen tissue was excellent as per the data sheet provided by the supplier (CHTN). The frozen tissues were kept at −80°C and paraffin blocked tissues stored at room temperature.

### Chemicals and reagents

Docetaxel, casodex, cyclopamine and cisplatin were purchased from LKT Laboratories (St. Paul, MN, USA). Puoromycin, G418 and BrdU labeling reagent were purchased from Invitrogen (Carlsbad, CA, USA). The anti-BMI1 antibody, ChIP-grade anti-TCF1 and anti-TCF4 antibody was obtained from Millipore (Bedford, MA, USA). Anti-BCL2 and anti-BrdU antibodies were purchased from Cell Signaling (Danvers, MA, USA).

### Transfections

Transfections were performed using Lipofectamine in CaP lines (LNCaP, PC3, Du145) and colon cancer cell line (HT29) (Invitrogen, Carlsbad, CA, USA) as per Vendor's protocols and as described earlier [19]–[20]. The effectiveness of transient transfections varied (65–85%) from cell line to cell line with the least (65%) in LNCaP and maximum (85%) in PC3 cells.

### Cell growth assay

Cell growth was determined by MTT (3-[4, 5-dimethylthiazol-2-yl]-2, 5-diphenyl tetrazoliumbromide; Sigma, Saint Louise, MO) assay as described earlier [19]–[20]. Briefly, transfected cells were grown in complete medium. Each condition was repeated in 10 wells. After incubation for specified time at 37°C in a humidified incubator, MTT (5 mg/ml in phosphate buffered saline, PBS) was added to each well. After 2 h of incubation with MTT, the plates were centrifuged at 500 *g* for 5 min. After careful removal of the solution, 0.1 ml of DMSO was added to each well and plates were shaken. The absorbance was recorded on a microplate reader at the wavelength of 540 nm. The cell growth was assessed as percent cell growth where vehicle-treated cells were taken as 100% viable.

### 3[H]-thymidine incorporation assay

3[H]-thymidine incorporation assay was performed as described earlier [19]. Briefly, Cells grown in 24-well plates in the presence of 3[H]thymidine (0.5 µCi/ml). Cells were then washed twice with cold PBS and then were incubated with trichloroacetic acid solution on ice for 30 min. Next, acid-insoluble fraction was dissolved in 1 ml of NaOH (1 M). Incorporated 3[H]thymidine were quantified using a scintillation counter.

### Colony formation assay

A total of 0.5% agar was prepared in appropriate culture media containing 20% fetal calf serum (bottom layer). Cells (1×10^5^ cell/100 mm plate) in 20% fetal calf serum and 0.7% agarose (top layer) were plated and incubated at 37°C. The medium was removed and replaced with fresh medium in every 2 days. After 14 days of incubation, the cells were stained with 0.05% crystal violet/methanol for 2 h and colonies were counted in two colony grids using a microscope.

### Immunohistochemistry

Immunostaining was performed as described earlier [20]–[21]. Briefly, paraffin embedded sections (to be evaluated for BMI1, BrdU and BCL2) were treated with Retrievagen A solution (pH 6) for antigen retrieval (BD Biosciences, San Diego, CA). Sections were incubated with primary antibody for overnight at 4°C. Slides were then washed and incubated for 2 h at room temperature with appropriate HRP-conjugated secondary antibody. Finally, slides were developed in 3, 3′-diaminobenzidene (DAB kit, Invitrogen) and counter stained with hematoxylin. The stained slides were dehydrated and mounted in permount solution.

### Western blot Analysis

Western blot analysis was performed as described earlier [19]–[21]. Briefly, cell lysates were prepared by incubation of cells for 30 min in ice-cold lysis buffer [(0.05 mmol/L Tris-HCl, 0.15 mmol/L NaCl, 1 mole/L EGTA, 1 mol/L EDTA, 20 mmol/L NaF, 100 mmol/L Na_3_VO4, 0.5% NP-40, 1% Triton X-100, 1 mol/L phenyl methylsulfonyl flouride (pH 7.4)] with protease inhibitor cocktail (Roche, Indianapolis, IN). The lysate was collected; insoluble materials were removed by centrifugation at 4°C for 15 minutes at 15,000 g, and stored at −80°C. BCA protein estimation kit was used to estimate the protein concentration in the lysates (Pierce, Rockford, IL), as per the vendor's protocol. Next, 40 µg protein was resolved in 10% SDS-PAGE gels, transferred onto PVDF membranes (Millipore) and incubated in blocking buffer (5% nonfat dry milk/1% Tween 20; in 20 mmol/L TBS, pH 7.6) for 2 h. The blots were incubated with appropriate primary antibody, washed and incubated with HRP-conjugated secondary antibody (Sigma). The blots were detected with chemiluminescence (ECL kit, Amersham Biosciences, Piscataway, NJ). Equal loading of protein was confirmed by stripping the blots and re-probing with ß-actin (Sigma).

### Luciferase reporter activity

In these studies, cells were co-transfected with the pTK-TCF, (200 ng/well) and pGeneClip-BMI1-shRNA or pbabe-BMI1. *Renilla* luciferase (pRL-TK) was used as an internal control and reporter activity was measured as described earlier [19]–[20]. For controls, the similar amount of empty vectors (pGL3, pbabe and pGeneClip) was transfected in cells.

### Quantification of apoptosis

Apoptosis was evaluated using the Annexin V-FITC Apoptosis detection kit from MBL International Corporation (Watertown, MA). Briefly, docetaxel resistant and BMI1-silenced docetaxel resistant cells were harvested with 0.025% trypsin + 5 mM EDTA in PBS (containing 2.5% FBS). Then the cells were washed with PBS and incubated for 5 min at room temperature with Annexin V-FITC plus propidium iodide (PI) as per vendor's protocol. Cells were analyzed on a Becton Dickinson FACS Calibur flow cytometer (BD Biosciences), placing the FITC signal in FL1 and the PI signal in FL2. Intact cells were gated in the FSC/SSC plot to exclude small debris. Cells in the lower right quadrant of the FL1/FL2 dot plot (labeled with Annexin V-FITC only) are considered to be in early apoptosis, and cells in the upper right quadrant (labeled with Annexin V-FITC and PI) are in late apoptosis/necrosis.

### Generation of stable cell lines

To generate BMI1-overexpressing and BMI1-silenced stable cells, PC3 cells were transfected with either pbabe-BMI1 or pGeneClip-BMI1-shRNA using Lipofectamine. BMI1 overexpressing cells were selected in presence of puromycin (1 µg/ml) and BMI1-silenced were selected in presence of G418 (400 µg/ml) starting at 48 h after transfection. The selection of cells under antibiotics was continued for 4-weeks and clones were tested for BMI1 expression. The stable BMI1-overexpressing and BMI1-slinencing clones were maintained in RPMI containing 10% FBS and respective antibiotics (0.5 µg/ml puromycin for overexpressing clones, and 300 µg/ml G418 for silenced clones). During stable cell selection, we obtained several clones which expressed BMI1 for different durations of time. We selected clones for our studies which exhibited the expression or suppression of BMI1 upto 4 months.

### Generation of chemoresistant cells

Chemoresistant cells were generated as per the method described by O'Neill et al. [22] (with modifications). Briefly, one million cells were seeded in 100 mm tissue culture dish with advanced-RPMI (Invitrogen) media containing 5% FBS and 1% penicillin/streptomycin for 24 h. At 24 h, the cells were exposed to 10 nM docetaxel for 48 h. The selection of concentration was based on the IC_50_ value. The drug containing medium was replaced after two days and the surviving (adherent) cells were cultured in a fresh drug-free complete medium. The cycle was repeated total 10 times. Following each treatment, cells were allowed to fully recover before next treatment. Next, the adherent cells were collected and exposed to higher doses of docetaxel (15, 20, to 25 nM) for 6 weeks (two weeks for each concentration). Finally, the surviving cells were maintained in RPMI/5% FBS containing 10 nM docetaxel. Untreated PC3 cells aged along side the treated cells (to avoid aging effect) were considered as control.

### RNA isolation and cDNA synthesis

Total RNA was isolated from cells in culture plates using Trizol reagent as per the vendors protocol (Invitrogen). 1 µg RNA was used to synthesize cDNA as described earlier [19].

### CaP-specific membrane hybridization and quantitative RT-PCR array

The membrane (printed with probes for CaP-specific genes) was hybridized with cRNA (synthesized from RNA of cells) and detected by chemiluminescence as per vendor's protocol (SA-Biosciences Super Array). Further, RNA from cells was used for qRT-PCR-array of CaP specific genes. The data analysis was performed by using array analyzer software (SA-Biosciences Super Array). The array data have been deposited to Gene bank database (accession number-GSE44049).

### Senescence-associated ß-Galactosidase analysis

ß-galactosidase activity was performed by using X-gal (5-bromo-4-chloro-3-indolyl-ß-d-galactopyranoside) kit (Cell Signaling) as per vendor's protocol.

### Chemosensitivity assay

Transfected cells at 12 h post-transfection were treated with casodex (10 µM), docetaxel (10 nM) and cisplatin (10 µM) for additional 24 h. Cells growth and proliferation were determined by 3[H]thymidine incorporation and colorimetric MTT-assay as described previously [19]–[20].

### Quantitative Chromatin Immunoprecipitation (ChIP) assay

ChIP analysis for TCF1 and TCF4 occupancy on promoter region of *BCL2* gene was performed as described [19]. Briefly, samples were cross-linked with 1% formaldehyde. Anti-AR antibodies were used with protein A-Sepharose (Sigma) to adsorb immune-specific complexes. Preimmune serum was used as a control. Purified DNA was analyzed by real-time PCR (ABI Prism 7500) using appropriate primers. Product was measured by SYBR green fluorescence and the amount of product was calculated by determining relative expression to a standard curve generated from a titration of input chromatin. Following primers were used to amplify segments that overlap with the appropriate regions: BCL2 (−3.91 Kb), Forward, *5′CTGTGGGAGCAAAGGAAGAC3′* ; Reverse, *5′AGAAGGAAACGGATCCCCTA3′* : BCL2 (P2-promoter, TATA site), Forward, *5′CAAGTGTTCCGCG’TGATTG3′* *;* Reverse *5′CCCGGTTA TCGTACCCTGTT3′* *:* BCL2 (−0.8 Kb), Forward, *5′GTCCAAGAATGCAAAGCACA3′* *;* Reverse*-* *5′CCCCCAGAGAAAGAAG AGGA3′* *;* SP5 (promoter) - Forward 5′-*GGGTCTCCAGGCGGC AAG-3* **′** **;** Reverse, *5′-AGCGAAAGCAAATCC TTTGAA-3′*.

### Tumor studies in animals

Athymic (nu/nu) male nude mice (6-weeks old) were implanted with stably transfected-PC3 cells (1×10^6^ in 50 µl RPMI +50 µl Matrigel) subcutaneously. The study comprised of two protocols. Animals under each protocol were further divided into four groups described as following:

#### Overexpression protocol

Group-1 (n = 10) implanted with empty-vector (pbabe) transfected cells and treated with vehicle served as control. Group-II (n = 10) implanted with vector transfected cells was treated docetaxel (10 mg/kg in 100 µl of saline; thrice/week) through intraperitoneal (*i.p*.) administration. Group-III (n = 10) implanted with BMI1-overexpressing cells received *i.p*. administration of saline (100 µl). Group-IV (n = 10) mice bearing BMI1-overexpressing tumors received docetaxel (thrice/week).

#### Silencing protocol

Group-1 (n = 10) implanted with empty-vector (pGenClip) transfected cells and treated with vehicle served as control. Group-II (n = 10) implanted with vector transfected cells was treated with docetaxel (10 mg/kg). Group-III (n = 10) implanted with BMI1-silenced cells received *i.p.* administration of saline. Group-IV (n = 10) implanted with BMI1-silenced cells received docetaxel.

#### Tumor measurement

Body weights and tumor growth were recorded weekly as described [19]–[20]. Tumors from three animals from each group/protocol were excised at the 35^th^ day post- administration when 100% of control animals reached the preset end-point tumor volume of 1000 mm^3^. Rest of the animals remained under protocol for a maximum time of 10 weeks. Before 2 h of sacrifice, each animal received an *i.p.* administration of BrdU (10 ml/kg) to label proliferating cells within tumors [20]. Animal were euthanized by a CO_2_ inhalation method. All procedures were approved by University of Minnesota Institutional Animal Care and Use Committee (IACUC). The approved IACUC protocol number from University of Minnesota is 1003A79141. All procedures were conducted in accordance with the IACUC guidelines.

### Statistical analyses

Student's *t test* for independent analysis was applied to evaluate differences between the treated and untreated cells. A Kaplan-Meier survival analysis with the corresponding Log-Rank and Linear Regression analysis was used to measure the rate of mean tumor volume growth as a function of time. Statistical analyses were carried out by using S-PLUS (Insightful, Seattle, WA). A p-value of <0.05 was considered to be statistically significant.

## Results

### BMI1 protein levels in human prostatic tissues increase with progressive stages of CaP

**Figure 1:**
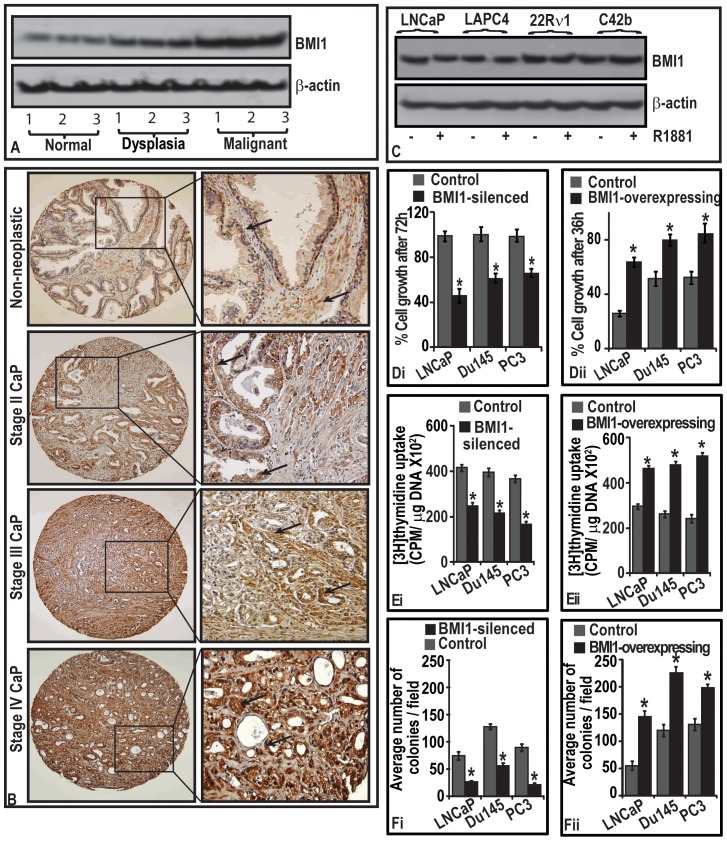
BMI1 protein levels are increased during the progression of CaP disease in human patients and BMI1 induces CaP cell proliferation. **(A)** Immunoblot represents BMI1 protein levels in normal, dysplasia and tumor prostatic tissues as assessed by immunoblotting **(B)** 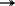 in representative photomicrographs point to BMI1-positive immunostaining in neoplastic and non-neoplastic regions of prostatic specimens. Magnification 40X. **(C)** Immunoblot represents the effect of androgen on BMI1 expression in cells assessed by immunoblotting. (Di–Dii; Ei–Eii and Fi–Fii) Histograms represent the growth, rate of proliferation and clonogenic proliferation of BMI1-silenced and -overexpressed CaP cells measured by MTT, 3[H]thymidine uptake and soft-agar colony formation assays. Each histogram represents mean ± S.E. of three independent experiments, * indicates p<0.05. Equal loading of protein for immunoblotting was confirmed by ß-actin.

As an attempt towards identifying the expression status of BMI1 in during progressive stages of CaP, we measured its levels by performing immunoblot analysis of human prostatic tissues from normal, dysplasia and CaP patients. Expression levels of BMI1 protein were higher in malignant than normal prostatic tissues (Figure 1A). We next determined the expression of BMI1 in human specimens of normal and CaP by employing immunohitochemical analysis. Immunostains showed granular cytoplasmic staining in both non-neoplastic and neoplastic epithelium. In general, the staining was stronger in neoplastic epithelial cells than in non-neoplastic epithelial cells (Figure 1B). A progressive increase of BMI1 in epithelial cells was observed as the disease progressed from low-grade to high-grade (Figure 1B). Recently, we observed that the staining pattern is stronger in neoplastic stroma than in non-neoplastic stroma of tissues from CaP patients [23]. Taken together, these data show that expression of BMI1 increases with increasing stage of CaP.

### BMI1 expression is independent of androgen

We investigated if the observed increase in BMI1 expression during the progression of CaP has a correlation with presence or absence of androgen. Androgen (R1881; 1 nM)-treated cells (LNCaP, LAPC4, 22Rν1 and C42b) did not show significant change in the levels of BMI1 protein (Figure 1C) suggesting that BMI1 expression is independent of influence of androgen.

### BMI1 regulates the proliferation of CaP cells

We investigated whether BMI1 regulates the growth of CaP cells. We employed a two-way approach where BMI1 was (i) either silenced by transfecting a vector-based pGenCLIP-BMI1-shRNA or (ii) overexpressed by transfecting pbabe-BMI1 plasmid in cells (LNCaP, Du145 and PC3) (Figure S1). As measured by MTT viability assay, LNCaP cells duplicate within 48–72 h while as Du145 and PC3 cells duplication takes 24 h *in vitro*. It is noteworthy that BMI1-silenced cells did grow only 50–65% even at 72 h post-transfection (Figure 1Di). However BMI1-overexpressing cells achieved 65–90% confluence only at 36 h post-transfection suggesting that presence of BMI1 has the significance in the growth of CaP cells (Figure 1Dii).

We next asked if the pro-growth role of BMI1 is due to its effect on the proliferative potential of tumor cells. The rate of 3[H]thymidine uptake by CaP cells showed that knockdown of BMI1 decreased the proliferation (Figure 1Ei). Conversely, overexpression of BMI1 significantly increased the rate of proliferation in cells (Figure 1Eii). Taking advantage of the use of vector-based shRNA that enabled us to investigate the effect of BMI1-silencing over a long period, we determined clonogenic proliferation of CaP cells. Suppression of BMI1 significantly reduced number of colonies formed in soft-agar. Conversely, BMI1-overexpressing CaP cells formed increased number of colonies suggesting that BMI1 confers proliferative attributes to tumor cells (Figure 1Fi–Fii).

### BMI1 increases the replicative life of normal cells

**Figure 2:**
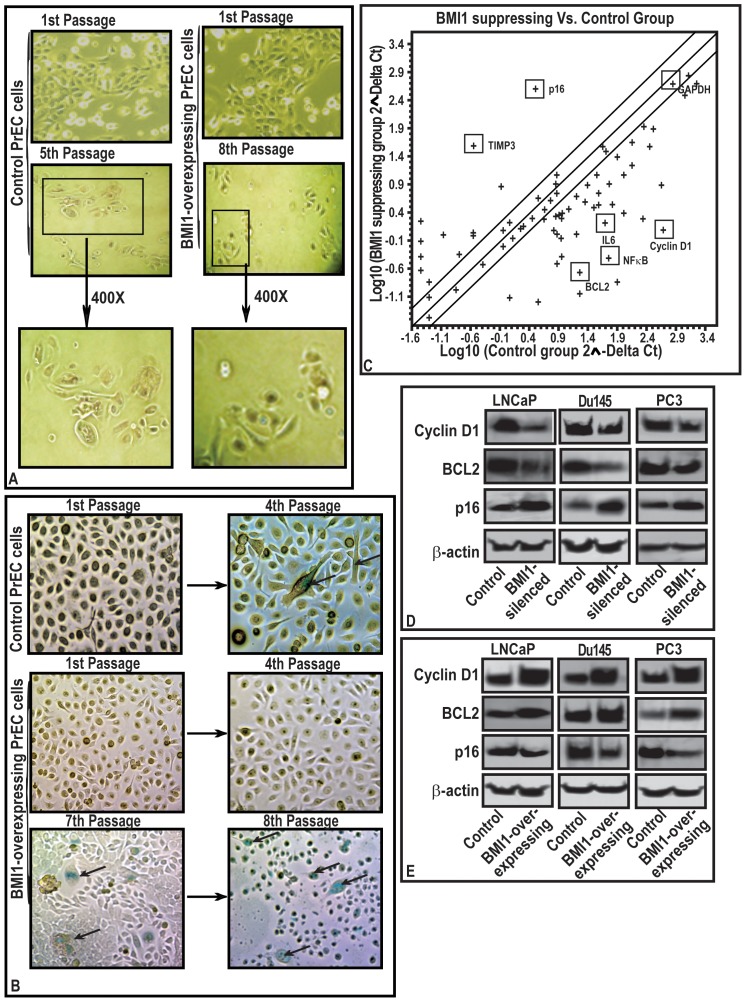
BMI1 induces growth of normal primary prostate cells (PrEC) by abolishing senescence and regulates the expression of proliferation-associated genes in CaP cells. **(A–B)** While PrEC replicated for 5 passages and entered into senescence, BMI1-rich counterparts replicated and avoided senescence upto 8th passages. (A) **Inset** 400X of magnified areas show senescent morphology features i.e. globular shape and (B) 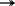 indicate staining for ß-galactosidase. **(C)** Scattered Plot for qPCR array. The dots indicate gene expression on a log-scale representing the change in BMI1 silenced-LNCaP cells. Fold change (2∧- Delta Ct) is the normalized expression (2∧-Delta Ct) in the BMI-silenced cells divided by the normalized expression of Control. **(D and E)** Immunoblots represents the effect of BMI1-silencing and BMI1-overexpression on the expression of Cyclin-D1, BCL2 and p16 proteins in cells. The equal loading of protein was confirmed by ß-actin.

Proliferation promoting properties of BMI1 compelled us to ask this protein drives the proliferation. For this purpose, we used primary prostate epithelial cells (PrEC). PrEC grow slowly and enter into senescence after 4–5 passages under culture conditions. Notably, forced expression of BMI1 caused an increase in the replicative cycles of normal PrEC (Figure 2A). Due to transient nature of transfection, the effect of overexpression lasted up to 8^th^ passage. Further, to validate senescence in PrEC, we measured senescence-associated β-galactosidase (SA-ß-gal) activity (marker of senescence). Normal cells exhibited positive staining for SA-ß-gal at the 4th passage. However BMI1-overexpressing PrEC did not show positive staining for SA-ß-gal at the 4^th^ passage, continued growing and exhibited SA-ß-gal activity at the 7^th^ passage (Figure 2B). These data show that BMI1 has the potential to drive normal as well tumor cells towards proliferation.

### Molecular mechanism of action of BMI1 in tumor cells

To investigate the mechanism through which BMI1 controls the proliferation, we performed CaP-focused membrane and qPCR-based array analysis (of genes involved in the proliferation). Since LNCaP cells exhibit the expression of majority of the human genes, we performed the primary analysis in these cells. A cut-out point of 2-fold was selected for analysis. After observing the effect of BMI1-silencing in LNCaP cells, the data was further validated in Du145 and PC3 cells (data not shown). BMI1-silenced LNCaP cells exhibited a significant change in the expression of several proliferation-associated genes (Figure 2C and Table S1). These included Cyclin-D1, BCL2, IL and NFκB (Figure 2C and Table S1). An increased expression of p16, p15 and TIMP3 in BMI1-silenced tumor cells was observed (Figure 2C and Table S1). The array data was validated by performing immunoblot analysis of selected gene products in CaP cells. BMI1-silenced cells exhibited decreased Cyclin-D1 and BCL2 and increased p16 protein levels (Figure 2D). These data were further validated by conducting RT-PCR based microarray study and immunoblotting of BMI1-overexpressing cells, which exhibited increased BCL2 and Cyclin-D1 levels (Figure 2E; Figure S2A). The data suggested a possible association between the BMI1, BCL2 and Cyclin-D1 in tumor cells.

### BMI1 in survival, growth and chemoresistance of tumor cells

**Figure 3:**
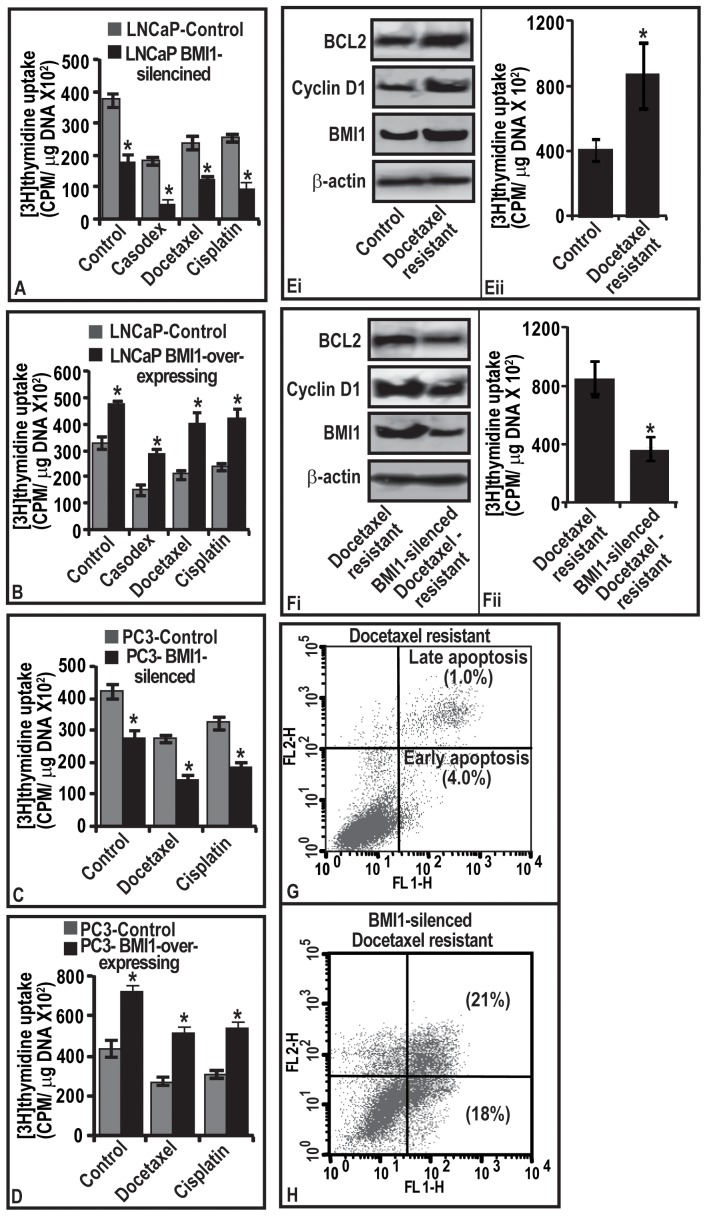
BMI1 confers chemoresistance to tumor cells. Rate of proliferation and apoptosis in cells were determined by 3[H]thymidine uptake and flow cytometery respectively. **(A–D)** Histograms represents the rate of proliferation in **(A–B)** LNCaP and **(C–D)** PC3 cells harboring varied BMI1 levels and treated with chemotherapeutic agents. Vehicle treated cells served as control. **(Ei and Fi)** immunoblots represent the levels of BMI1, Cyclin-D1 and BCL2 proteins in docetaxel-resistant, and BMI1-silenced docetaxel-resistant cells. (Eii and Fii) Histograms showing the rate of proliferation in docetaxel-resistant, and BMI1-silenced docetaxel-resistant cells. For immunoblot analyses (Figure Ei and Fi), equal loading of proteins was confirmed by ß-actin. (A–D, Eii and Fii) Each bar represents mean ± SE of three independent experiments, * represents P<0.05. **(G–H)** represents quantitative estimation of apoptosis in BMI1-silencing chemoresistant cells. The lower right quadrant of the FL1/FL2 plot (Annexin V-FITC) represent early apoptosis and the upper right quadrant (labeled with AnnexinV-FITC and PI) represent late apoptosis.

Since BMI1 was observed to regulate the expression of proliferation-associated genes, we sought to determine if this phenomenon is responsible for chemoresistance. We determined the growth potential of BMI1-overexpressing and BMI1-silencing CaP cells treated with clinically used chemotherapeutic agents (bicalutamide/casodex, docetaxel and cisplatin). The selection of dose of chemotherapeutic agents was based on their growth-inhibitory potential against LNCaP cells in a time and dose-dependent study (Figure S2C–D). The 3[H]thymidine incorporation analyses showed that BMI1-overexpressing CaP cells proliferate at high rate and are non-responsive to drugs (Figure 3B and D). However, BMI1-silenced cells were responsive to drugs and exhibited reduced rate of proliferation (Figure 3A and C). The observed differences between the BMI-overexpressing and -silenced tumor cells in responsiveness towards chemotherapy were also reflected in cell viability (Figures S3 and S4). These data suggest that presence of BMI1 in tumor cells plays a critical role in deciding the therapeutic outcome and could be clinically relevant.

### BMI1 is critical for regrowth of tumors post-chemotherapy

We conducted a proof of principle study with chemoresistant PC3 cells. We showed that chemoresistant PC3 cells exhibited increased growth and expression of BMI1, BCL2 and Cyclin-D1 (Figure 3Ei–ii **)**. We asked if targeting BMI1 could render the chemoresistant cells amenable to therapy and guide these to apoptosis. As evident from 3[H]thymidine uptake and FACS-analysis data, knocking-down of BMI1 inhibited the proliferation and increased apoptosis of chemoresistant cells concomitant to a decrease in BCL2/Cyclin-D1 levels (Figure 3F–H). These data establish role of BMI1 in the recurrence of tumor cells post-chemotherapy.

### Molecular mechanism through which BMI1 regulates BCL2 in cells

**Figure 4:**
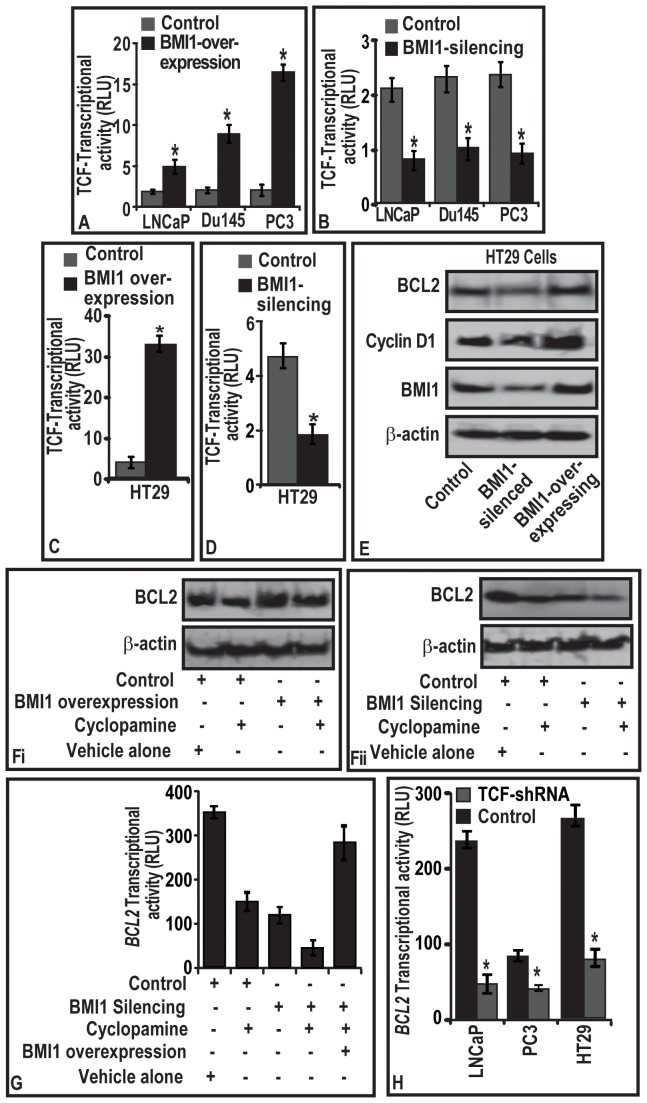
BMI1 regulates BCL2 expression through activation of TCF-transcriptional activity in tumor cells. **(A–B; C–D)** Histograms represent the effect of BMI1-overexpression and BM1-silencing on the transcriptional activation of *TCF-responsive element* in CaP and HT29 cells cells as assessed by luciferase-reporter assays. **(E–F)** representative immunoblots showing the effect of BMI1-silencing and -overexpression on the levels of BCL2 and Cyclin-D1 proteins in **(E)** HT29 cells, and (Fi–Fii) CaP cells treated with Cyclopamine (Shh inhibitor) for 12 h. Control cells were treated with DMSO. (G–H) Histogram represents the effect of **(G)** cyclopamine treatment and (H) TCF silencing on the transcriptional activity of BCL2 promoter in LNCaP, PC3 and HT29 cells. (A–D; G–H), relative luciferase activities were calculated with the values from vector group, and each bar represents mean ± SE of three independent experiments, *represents p<0.05. **(E–F)** Equal loading of proteins was confirmed by testing immunoblots for ß-actin.

Cyclin-D1 and BCL2 are down-stream targets of Wnt and Sonic hedgehog (Shh) signaling [24]–[25]. Therefore, we asked if BMI1 (i) has any association with Wnt and Shh-signaling in proliferating cells, and (ii) regulates BCL2 under Wnt or Shh guidance. We first determined the status of Wnt-signaling in BMI1-silenced and BMI1-overexpressing cells. By utilizing the reporter activity of *TCF-responsive element* (biomarker for Wnt activation), we observed that BMI1-overexpressed cells harbor increased TCF-transcriptional activity concomitant with the increased BCL2 and Cyclin-D1 (Figure 4A). Notably, BMI1-silenced cells exhibited decreased TCF-transcriptional activation (Figure 4B). Since our data showing a regulatory role of BMI1 on TCF-transcriptional activity in tumor cells is novel, we asked if this phenomenon is limited to CaP cells. To validate, we selected colon-cancer cell-line HT29 (exhibits increased Wnt signaling) and expresses BMI1 [26]. Interestingly, either BMI1-silencing or BMI1-overexpression caused significant modulations of TCF-transcriptional activity concomitant with changes in BCL2 and Cyclin-D1 in HT29 cells. These data establish the regulatory role of BMI1 on TCF-transcriptional activity in other cell types (Figure 4C–E).

BCL2 is a well-established downstream target of Shh signaling [27]. We asked if BMI1 (i) partially regulates BCL2, or (ii) is regulation of BCL2 solely under the control of Shh? For this reason, cyclopamine (5 µM), an inhibitor of Shh-signaling was used. As expected cyclopamine treatment caused a reduction in BCL2 in control cells, however failed to modulate BCL2 levels in BMI1-overexpressing cells (Figure 4Fi). Notably, targeted-knockdown of BMI1 caused cyclopamine-resistant BMI1-rich cells to respond well to cyclopamine and reduced BCL2 (Figure 4Fii). To further validate the Shh-independent role of BMI1 in regulating BCL2 expression, we next reintroduced BMI1 in (a) cyclopamine treated, and (b) BMI1-silenced cells. Reintroduction of BMI1 in cyclopamine treated and BMI1-silenced cells caused a gain in BCL2 promoter-activity (Figure 4G). Taken together these data (Figure 4F–G) suggest that BCL2 expression is regulated in part by BMI1 and partially by Shh-signaling.

Since both TCF and BCL2 were observed to be under the guidance of BMI1, we next investigated whether there is a direct association of TCF and BCL2 gene in tumor cells. CaP and HT29 cells were co-transfected with TCF-shRNA and BCL2-luc reporter and were analyzed for BCL2-promoter activity. TCF-silenced CaP and HT29 cells exhibited decreased BCL2-promoter-activity (Figure 4H). This report suggests that BCL2 acts as a downstream target of TCF-signaling.

### TCF4-transcriptional factor binds to promoter region of BCL2 gene in tumor cells

**Figure 5:**
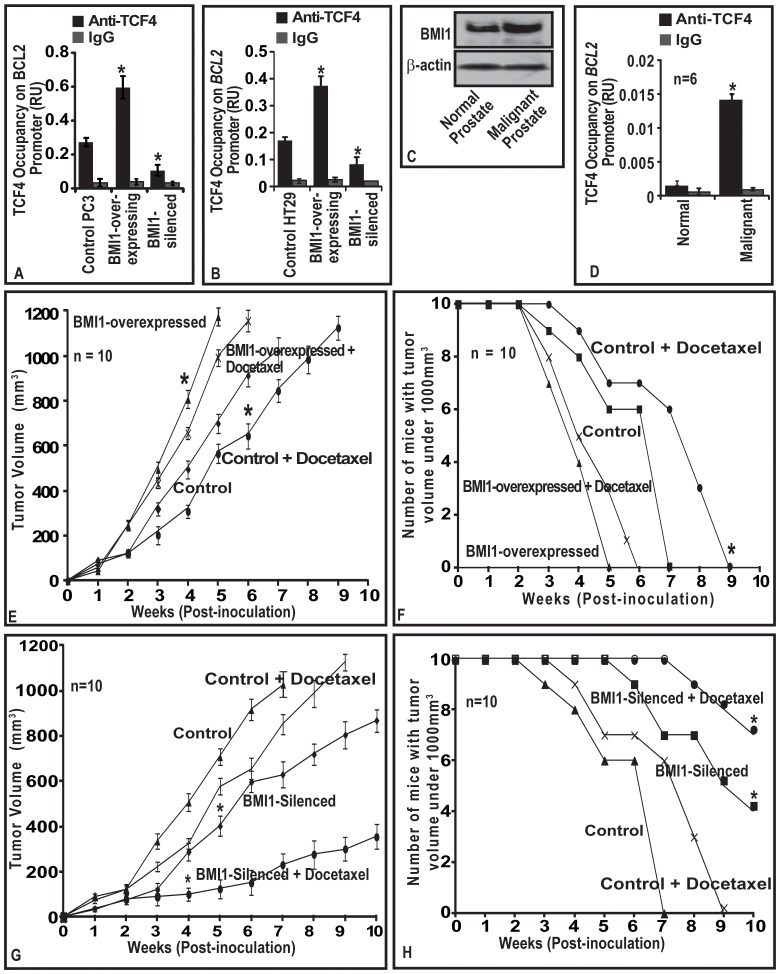
(A–B) BMI1 induces TCF4 binding to promoter region of *BCL2* gene. (C) TCF4 occupancy on *BCL2* is elevated in malignant prostatic tissues. (D) BMI1 confers chemoresistance to tumors in a mouse model. **(A–B)** Histogram represents effect of BMI1 expression on TCF4-occupancy on promoter regions of *BCL2* in PC-3 and HT29 cells as assessed by ChIP assay. **(C–D)** immunoblot and histogram represents the BMI1 protein expression, and TCF4-occupancy on *BCl2 gene* in normal and malignant human prostate tissues as assessed by immunoblotting and ChIP assays. Equal loading of proteins was confirmed by ß-actin for immunoblotting. (A–B, D). Each bar represents mean ± SE of three independent experiments. **(E–F)** The line graph represents average volume of BMI1-overexpressing and BMI1-suppressed tumors as a function of time vis-à-vis docetaxel therapy on in nude mice. **(G–H)** The line graph shows the number of mice with tumor volumes <1000 mm^3^ for indicated weeks. Data is represented as mean±SE; * indicates p<0.05.

To further identify the underlying mechanism, we investigated whether BCL2 gene has possible TCF binding sites on its promoter. By employing TESS analysis, we observed that BCL2-promoter region exhibits multiple sites where TCF possess the affinity to bind (data not shown) [28]. We next sought the validation of TESS data (which is a mathematical data) by biochemical analysis. We tested TCF1 and TCF4 occupancy on multiple sites on the promoter region of BCL2 gene by employing ChIP assay. The TCF unresponsive SP5-promoter was used as a negative control (data not shown). TCF1 was observed to have insignificant occupancy on the examined sites of BCL2-promoter in both CaP and colon cells (data not shown). Among the three region analyzed, TCF4 was observed to occupy only TATA region of BCL2-promoter (P2-promoter). We found very little or no occupancy by TCF4 on −3.41 Kb and −8.41 kb of BCL2 promoter (data not shown). Notably, BMI1-overexpression was observed to increase the TCF4 occupancy on the TATA region of BCL2-promoter in the PC3 and HT29 cells (Figure 5A–B). An opposite result was observed with that of BMI1-silenced CaP and HT29 cells (Figure 5A–B).

### TCF4 binds to promoter region of BCL2 gene in human prostatic tissues

We next investigated if the *in vitro* observation of TCF4-occupancy on BCL2-promoter binding has translational relevance. From the outcome of immunoblot analysis data (Figure 5C), we selected human normal and malignant prostatic tissues (which exhibited increased BMI1) and determined TCF4-occupancy on *BCL2-*promoter. As compared to the normal prostatic tissues, TCF4 exhibited increased occupancy at TATA region of *BCL2* in human CaP tissues (Figure 5D).

### BMI1 confers chemoresistance to human prostatic tumors in xenograft mouse models

Since BMI1 was observed to be involved in the chemoresistance of CaP cells to various antitumor agents under *in vitro* conditions, we next determined whether these observations could be translated under *in vivo*. For this purpose, we measured the differential growth rate of tumors-derived from BMI1-overexpressing and –silencing PC3-stable cells under two protocols.

#### BMI1-overexpression protocol

At one week post-implantation, the average volume of control and BMI1-overexpressing tumors in mice increased as a function of time (Figure 5E). BMI1-overexpressing tumors were observed to grow at faster rate and larger in size than control tumors (Figure 5E). Mice implanted with control-tumors reached a preset end-point tumor volume of 1000 mm3 at 49th day of post-implantation (Figure 5E). However, mice implanted with BMI1-overexpressing tumors reached the preset end-point tumor volume at 35th day of post-implantation (Figure 5E). At 49th and 35th days, mice implanted with control and BMI1-overexpressing tumors exhibited average tumor volumes of 1076 and 1023 mm3, respectively (Figure 5E).

Docetaxel treatment decreased the growth of tumors in control mice (Group-II). Interestingly, docetaxel treatment failed (for 10-weeks) to produce an effect on the growth of tumors expressing high BMI1 protein. At 49^th^ day, the average tumor volume in control mice (Group-II) treated with docetaxel was 850 mm^3^ (Figure 5E). However, BMI1-overexpressing tumors though treated with docetaxel reached an average tumor volume of 997 mm^3^ at 35^th^ day (Figure 5E). Next, we evaluated whether treatment of docetaxel to animals caused a delay in the growth of tumors harboring increased BMI1 levels. As evident from the number of mice reaching the preset end-point tumor volume (1000 mm^3^), the observed differences between the control and BMI1-overexpresed group of animals for the outcome of docetaxel therapy was statistically significant (Figure 5F).

#### BMI1-silenced protocol

BMI1-silenced cell-derived tumors were observed to grow at slower rate than control (Figure 5G). This was evident from the significant difference in the rate of growth and tumor volumes between control and BMI1-silenced groups (Figure 5G). Control group reached a preset end-point average tumor volume of 1000 mm^3^ at 49^th^ day post-implantation (Figure 5G). Notably BMI1-silenced group did not reach the end-point even at 70^th^ day post-implantation (Figure 5G). At 49^th^ day, animals treated with docetaxel exhibited an average tumor volume of 850 mm^3^. However at this point, BMI1-silenced group treated with docetaxel exhibited only an average tumor volume of 230 mm^3^ suggesting that knocking down of BMI1 sensitized tumor cells to therapy (Figure 5G). Further docetaxel caused a delay in the growth of BMI1-silenced tumors. The observed differences between control and BMI-silenced group were significant (Figure 5H).

## Discussion

Recent studies showed that dysregulation of BMI1 play a crucial role in epithelial mesenchymal transition, cell proliferation, senescence and self-renewability of several human cancers [11], [16], [29]. However, the role of BMI1 in CaP progression and chemoresistance is not well studied [30]. It is speculated that inability of tumor cells to undergo apoptosis in response to chemotherapy results in a selective advantage for such cells to become more aggressive compared to chemoresponsive cells [11]. Although several studies demonstrate that BMI1 rescues tumor cells from apoptosis, the concrete mechanism of action is yet to be known [13]–[15]. CRPC is hard-to-treat disease and identifying a critical molecule that confers the chemoresistant characteristic to such tumors would be an important advancement in this field. In the current study, we provide mechanism-based evidence that BMI1 plays a deciding role in the fate of tumor cells undergoing chemotherapy. This study is significant because we demonstrated that BMI1 equally confers chemoresistance to hormone-sensitive CaP and CRPC cells. CRPC tumors in men are reported to proliferate under low androgen conditions [7], [31]. An important clinically relevant information from this study is that BMI1 expression does not get influenced by androgen suggesting a possibility for BMI1 playing a role in driving indolent disease to aggressive androgen-independent phenotype. Based on our data we suggest that targeting BMI1 should be a part of therapeutic strategy to combat chemoresistant cancer.

Expression of BCL2 and Cyclin-D1 is reported to be high in chemoresistant tumors [24], [32]–[34]. Notably, BCL2 and Cyclin-D1 have a commonality to be also the functional members of Wnt and Shh pathways. Recent studies suggest that targeting BCL2 directly (anti-BCL2 immunotherapy) or blocking the pathways (such as Shh) regulating it, could be a possible therapeutic strategy to overcome chemoresistance [30], [35]–[36]. Shh inhibitors (which downregulate BCL2) are currently being investigated as therapeutic agents for basal cell carcinoma, medulloblastoma and glioblastoma [37]. Despite treated with anti-BCL2 therapies, the tumors resurge for unknown reasons. Our study is significant because we provide evidence that BCL2 is not completely lost in chemoresistant tumor cells post-chemotherapy, and alternate pathways regulate BCL2. This is based on data that (i) BMI1 regulates BCL2 independent of Shh-signaling and (ii) elevated levels of BMI1 and BCL2 are found in cells those escape chemotherapy. We suggest that this mechanism could be an explanation for the survival of chemoresistant cells post-chemotherapy. Although the previous report showed that BMI1 itself is a target of Shh-signaling, our data show that BMI1 acts independent of Shh [38]. It is possible that chemoresistant cells expressing BMI1 are a highly selected sub-population that remains hard to treat and play an important role in indolence of disease in CaP patients.

BMI1 activity is manifested in the form of repression of target genes and the mode of action could be through epigenetic silencing [11]. However, in this study we observed that BMI1-upregulates BCL2. Keeping in view the repressive nature of BMI1, there was a need to understand the mode of action through which BMI1 induces BCL2 activity. We provided evidence that BCL2 activation in chemoresistant cells under the guidance of BMI1 is mediated by TCF4. This was validated in prostate and colon cancer cells; and in human prostatic tissues. Although the complete information about the regulation of TCF4 by BMI1 is not completely understood, current data suggest that TCF4 indeed is in part under the control of BMI1. The significance of our data is that it (***i***) identifies BMI1-induced TCF4 as a molecular module that drives Wnt-signaling within chemoresistant cells, and (***ii***) BCl2 as a target of BMI1/TCF4 molecular module. Based on our data, we speculate that molecular module could be operational during emergence of chemoresistance and also responsible for proliferation of chemoresistant CaP cells after therapy.

Docetaxel has been tested under several clinical trials alone and in combination with other agents to treat CaP. Docetaxel therapy was observed to result in a PSA drop of more than 50% in CaP patients, an observation made in several trials such as the SWOG trial [31]. However, docetaxel alone, and in combination do not completely abrogate the tumor or bring down PSA levels to the normal in human CaP patients [39]. Although effective in CaP patients to an extent, some CaP conditions do not respond to docetaxel therapy [40]. Resistance to docetaxel is explained in part, by over-expression of deferent multidrug resistance proteins at both genetic and epigenetic levels [41]–[42]. In this context, this study is significant as we show that targeting BMI1 in chemoresistant cells sensitizes cells to therapy. This study identified BMI1 as an ideal molecule to be targeted to overcome the chemoresistance of CaP cells and corroborates to earlier report showing the utility of BMI1 as a target to overcome chemoresistance in ovarian cancer cells [15]. Under *in vivo* conditions, the significance between BMI1-positive, BMI-silenced and BMI1-overexpressed tumor cells vis-à-vis docetaxel therapy was significant. The success of docetaxel therapy against prostatic tumors in a xenograft mouse model was observed to be highly dependent on the level of BMI1. We suggest that preventing the development of chemoresistance in CaP patients will be beneficial for a large group of patients and interventions directed against BMI1 may provide opportunities to enhance the efficacy of chemotherapy. In this direction we have opened another front by identifying small molecule inhibitors (SMIs) of BMI1. We suggest that these should be explored against chemoresistant tumors. The advanced work with SMIs of BMI1 against chemoresistant tumors is underway in our laboratory.

## Supporting Information

Figure S1 **Representative immunoblot shows the effect of BMI1-silencing and -overexpression on the level of BMI1 protein in CaP cells.** Equal loading was confirmed by reprobing immunoblots for ß-actin.
(TIF)

Figure S2 **BMI1 modulates number of proliferation related genes in CaP cells and time-course and dose titration curves for the chemotherapeutic drugs.** **(A)** Figure represents clustrogram of gene expression as assessed by PCR array in control and BMI1-silenced LNCaP cells. **(B–D)** Time-course and dose titration curves for the chemotherapeutic agents Casodex **(B)**, Cisplatin **(C)** and Docetaxel **(D)** as assessed by MTT assay. Vehicle treated cells were considered as control. Data represents mean ± SE of three independent experiments.
(TIF)

Figure S3 **BMI1 regulates the growth of CaP cells. BMI1-rich CaP cells exhibit increased growth and chemoresistant against chemotherapeutic drugs.** **(A–B)** The histogram represents the rate of proliferation of cells as measured by MTT assay in BMI1 overexpressing **(A)** LNCaP and **(B)** PC3 cells treated with different chemotherapeutic agents. Vehicle treated cells were considered as control. Each bar in the histogram, represents mean ± SE of three independent experiments, * represents P<0.05.
(TIF)

Figure S4 **BMI1 regulates the growth of CaP cells. BMI1-deficient CaP cells exhibit decreased growth and chemo-sensitivity against chemotherapeutic drugs.** **(A–B)** The histogram represents the rate of proliferation of cells as measured by MTT assay in BMI1-silenced **(A)** LNCaP and **(B)** PC3 cells treated with different chemotherapeutic agents. Vehicle treated cells were considered as control. Each bar in the histogram, represents mean ± SE of three independent experiments, * represents P<0.05.
(TIF)

Table S1 List of selected genes modulated by BMI1-supression in CaP cells.
(DOC)
