# Supplementary figures and images for: BMI1 Polycomb Group Protein Acts as a Master Switch for Growth and Death of Tumor Cells: Regulates TCF4-Transcriptional Factor-Induced BCL2 Signaling
Source: PLoS One. 2013 May 6;8(5):e60664. doi: 10.1371/journal.pone.0060664 (PMC3645992; doi:10.1371/journal.pone.0060664)

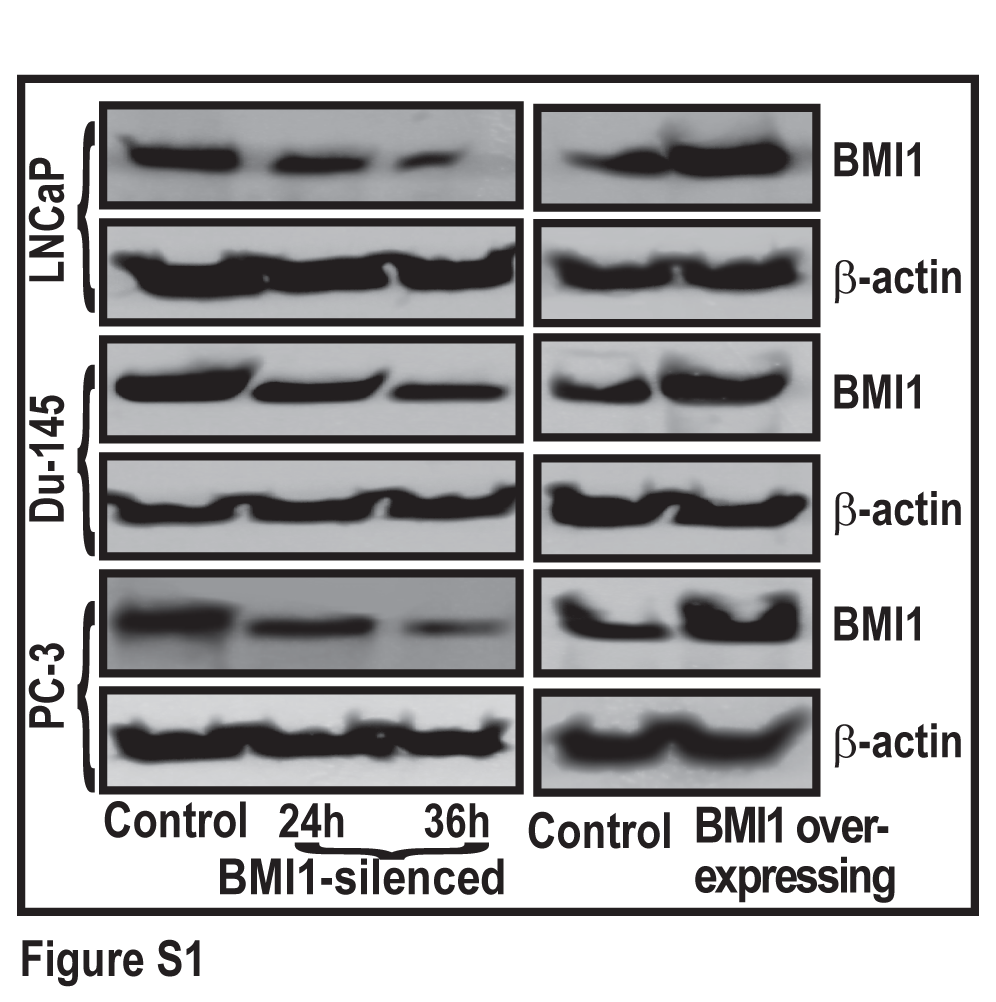

Supplement: Figure S1 — Representative immunoblot shows the effect of BMI1-silencing and -overexpression on the level of BMI1 protein in CaP cells. Equal loading was confirmed by reprobing immunoblots for ß-actin. (TIF) [file pone.0060664.s001.tif]

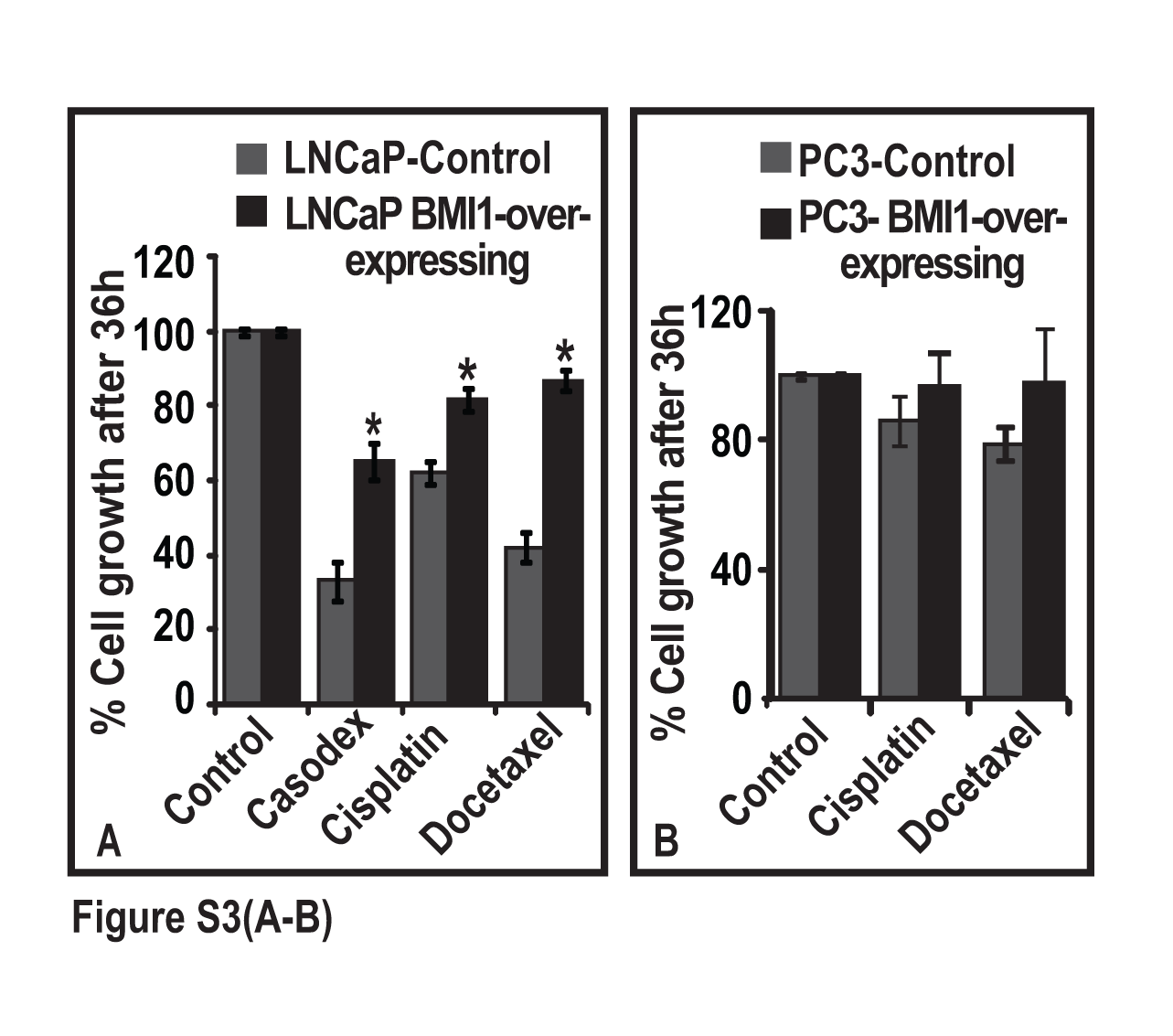

Supplement: Figure S3 — BMI1 regulates the growth of CaP cells. BMI1-rich CaP cells exhibit increased growth and chemoresistant against chemotherapeutic drugs. (A–B) The histogram represents the rate of proliferation of cells as measured by MTT assay in BMI1 overexpressing (A) LNCaP and (B) PC3 cells treated with different chemotherapeutic agents. Vehicle treated cells were considered as control. Each bar in the histogram, represents mean ± SE of three independent experiments, * represents P<0.05. (TIF) [file pone.0060664.s003.tif]

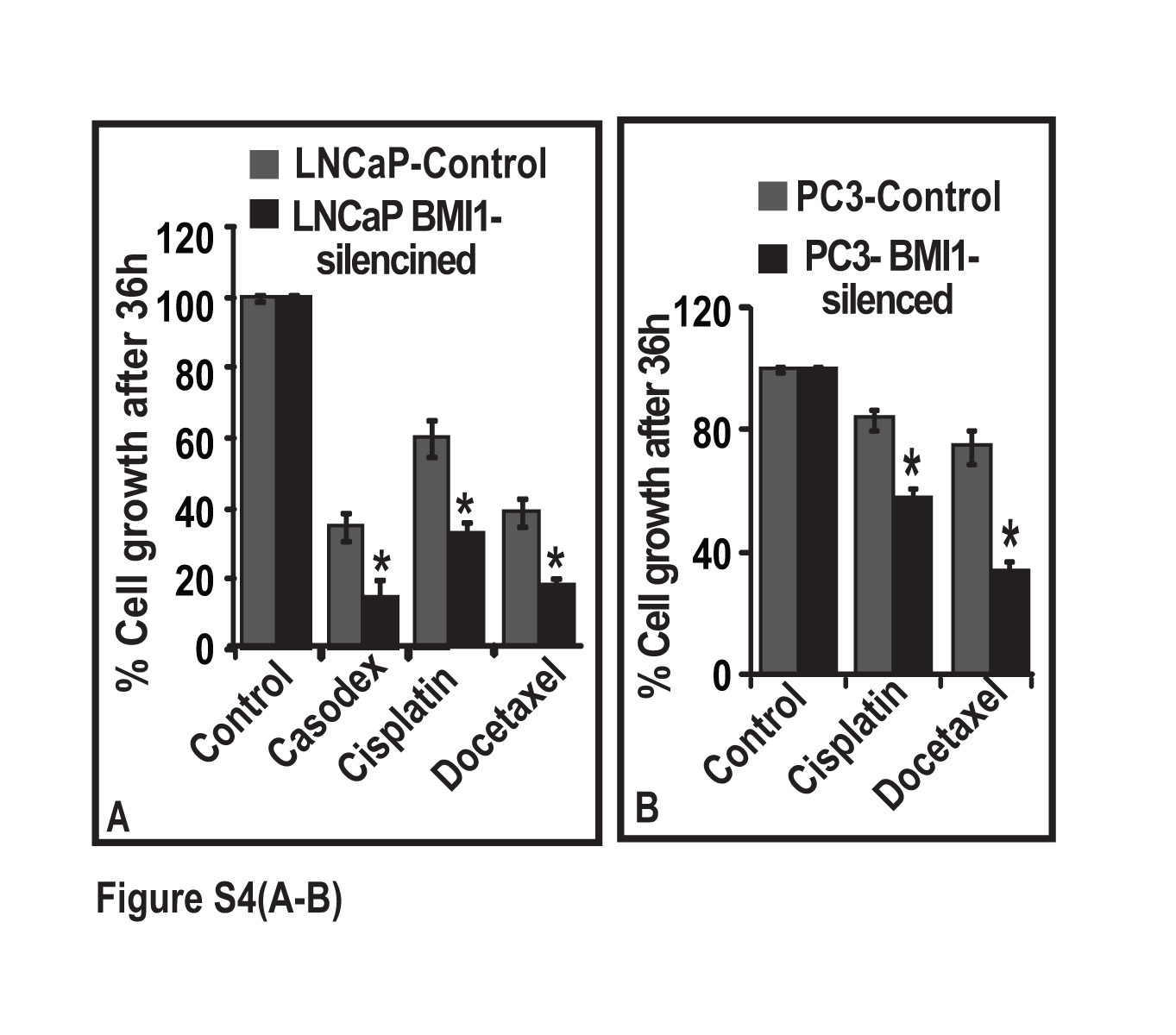

Supplement: Figure S4 — BMI1 regulates the growth of CaP cells. BMI1-deficient CaP cells exhibit decreased growth and chemo-sensitivity against chemotherapeutic drugs. (A–B) The histogram represents the rate of proliferation of cells as measured by MTT assay in BMI1-silenced (A) LNCaP and (B) PC3 cells treated with different chemotherapeutic agents. Vehicle treated cells were considered as control. Each bar in the histogram, represents mean ± SE of three independent experiments, * represents P<0.05. (TIF) [file pone.0060664.s004.tif]
