# Supplementary material for: BMI1 Polycomb Group Protein Acts as a Master Switch for Growth and Death of Tumor Cells: Regulates TCF4-Transcriptional Factor-Induced BCL2 Signaling
Source: PLoS One. 2013 May 6;8(5):e60664. doi: 10.1371/journal.pone.0060664 (PMC3645992; doi:10.1371/journal.pone.0060664)
Supplement: Table S1 — List of selected genes modulated by BMI1-supression in CaP cells. (DOC) [file pone.0060664.s005.doc]

| **Gene Symbol** | **Fold Changes** | **Function** | **Potential implication in CaP pathogenesis** |
| --- | --- | --- | --- |
| **Downregulated** |  |  |  |
| Cyclin D1 | 26.0 | Cell cycle, cell growth and/or maintenance | Cell proliferation |
| Cdk4 | 7.5 | Regulation of cell cycle; protein Kinase activity | Cell Proliferation |
| Bcl-2 | 20.0 | Mitochondrial protein that blocks the apoptotic death | Cell proliferation and chemoresistance |
| Akt-1 | 3.5 | Survival factors; suppress apoptosis | Cell proliferation and chemoresistance |
| PI3K | 3.0 | Pro-survival factor | Cell Proliferation |
| u-PA | 6.5 | Involved in degradation of the extracellular matrix | Cell migration and proliferation |
| Erbb2 | 7.0 | Kinase activity, phosphorylation, receptor activity | Cell proliferation and tumorigenesis |
| Src | 2.5 | Regulation of cell growth and development | Cancer progression |
| c-Myc | 8.5 | Transcription factor | Cell proliferation and tumorigenesis |
| c-Jun | 2.5 | Transcription factor | Cell survival and tumorigenesis |
| H-Ras | 2.0 | Signal transduction | Tumorigenesis |
| VEGF | 6.5 | Growth factor | Promoting cell migration, and inhibiting apoptosis |
| MMP-9 | 11.0 | Endopeptidases degrades extracellular matrix | Metastasis |
| NFκB1 | 4.0 | Signal transduction, transcription factor | Survival, invasion and chemoresistance |
| HIF-1 | 3.0 | Transcription factor | Tumor angiogenesis |
| IGF1 | 8.0 | Growth factor receptor binds insulin-like growth factors | Cell survival and tumorigenesis |
| IGF2 | 9.0 | Growth factor receptor binds insulin-like growth factors | Tumorigenesis |
| IL-2 | 10.0 | Inflammation response, cell proliferation | Inflammation, cell growth |
| GGT2 | 3.5 | Glutathione homeostasis | Tumorigenesis |
| PKC δ | 2.5 | Cell signaling, phosphorylation | Cell growth |
| PKCε | 2.0 | Cell Signaling | Cell growth, tumorigenesis |
| **Upregulated** |  |  |  |
| p16/Ink4 | 22.0 | Tumor suppressor, cell-cycle arrest in G1 & G2 phase | Proliferation inhibition |
| p15 | 9.0 | Cell growth regulator, controls cell cycle | Growth inhibition |
| p57 | 10.0 | Stem cell localization to the bone marrow | Proliferation inhibition |
| CD164 | 3.0 | Negatively regulating cell proliferation | Negative regulator of cell proliferation |
| Cadherin-9 | 4.0 | Calcium-dependent cell-cell adhesion | Invasion and metastasis inhibition |
| TIMP-3 | 2.5 | Metalloendopeptidase inhibitor activity | Induces apoptosis, inhibits invasion |
| PIas2 | 2.0 | Sumoylation | Cytokine inhibitors, Inflammation inhibitors |

**Table S1. List of Selected Genes Modulated by BMI1-supression in CaP Cells.**
